# Archival data on wild food plants used in Poland in 1948

**DOI:** 10.1186/1746-4269-4-4

**Published:** 2008-01-24

**Authors:** Łukasz Łuczaj

**Affiliations:** 1 High School of Humanities and Economics in Łódź, Department of Humanities, ul. Rewolucji 1905 r. nr 64, 90-222 Łódź, Poland; 2 Rzepnik 20A, 38-471 Wojaszówka, Poland

## Abstract

**Background:**

In 1948, Professor Józef Gajek initiated a detailed census of the wild edible plants used in Poland. The questionnaires were collected by correspondents of the Polish Folklore Society in 95 localities throughout Poland. A major part of these archival materials, including a substantial collection of herbarium specimens, had not undergone thorough analysis prior to this study, which presents a quantitative analysis of this archival set of data.

**Methods:**

Herbarium specimens were identified and a database was created.

**Results:**

Ninety-eight taxa identified to genus or species level, including 71 botanical species, identified using herbarium specimens, were found. On average only 11 edible plant species per locality were listed, the longest list included 39 species. No correlation between latitude and the number of edible species was found, whereas there was small but significant correlation with the longitude. Fruits were the most frequently collected part of plants. Most plants were primarily collected by women and children. Children both helped parents to collect wild fruits and also ate many species raw, which were not consumed by adults, but had often been eaten in the past. Eighteen of the taxa had not been reported in a recent comprehensive review of edible plants of Poland. *Stratiotes aloides*, used as a famine vegetable in the Łódź region, has never been reported as edible in any ethnobotanical literature.

**Conclusion:**

The results undermine the conclusions of a recent comprehensive review of edible plants of Poland, which stated that many more wild edible plants have been collected in the Carpathians than in lowland Poland. However such results were shown to be caused by the substantially larger number of ethnographic studies undertaken in the Carpathians. In fact, large numbers of edible plant species were collected in the mid-20^th ^century in a few regions, particularly along the eastern border, in the Carpathians and in communities originating from the expanded Soviet Union, which had been resettled to the north-west of Poland in 1945.

## Background

Łuczaj & Szymański recently published a review of the literature concerning wild edible plants of Poland, including a list of species which have been consumed in Poland over the last 200 years [1]. During the literature search for this review, vast amounts of unpublished archival material on the gathering of wild plants were discovered (stored in universities, museums, the Polish Folklore Society in Wrocław, and the office of the Ethnographic Atlas of Poland in Cieszyn) in the form of questionnaires and field notes from various ethnographic studies [1]. The main problem encountered in the analysis of such archival ethnographic descriptions (both published and unpublished) is the lack of corresponding herbarium specimens enabling the verification of plant identification. However, one set of data was found which did not have this flaw and was richly documented by dried plant specimens, constituting one of the most important ethnobotanical sources in Poland. It was a set of questionnaires from the Polish Ethnographic Atlas, 1948, stored in the Polish Ethnographic Atlas office in the University of Silesia (Cieszyn), with a small subset found in the archive of the Institute of Ethnology and Cultural Anthropology of the Jagiellonian University in Kraków, stored as "Odpowiedzi na ankietę nadesłane przez Koła Krajoznawcze Młodzieży Szkolnej", archive no. KKMS 317–332.

The Polish Ethnographic Atlas is unique among European ethnographic atlases, in its extensive coverage of many ethnobotanical topics. This large-scale ethnobotanical research was initiated and carried out by its first director, Józef Gajek, and then continued by his successors Janusz Bohdanowicz and Zygmunt Kłodnicki [2-5]. Although the undertaking of the Atlas was to describe all aspects of Polish folklore, its first four questionnaires concerned the use of wild edible plants (Questionnaires 1 and 2) and medicinal plants (Questionnaires 3 and 4) only. These four questionnaires were used together. They were filled in by a range of correspondents of the Polish Folklore Society (*Polskie Towarzystwo Ludoznawcze*), who interviewed local people, and sent the results back to the Polish Ethnographic Atlas office. In this study only Questionnaires 1 and 2 were analysed.

Questionnaire 1 was an empty table with two columns, one for local plant names and the other for the plant part used. Questionnaire 2 was used to provide more information on particular species, so questions about each species occupied two pages, including a space in which to attach a small herbarium specimen (Table 1). In reality some respondents sent both Questionnaire 1 and 2, and some only Questionnaire 1 or only 2, so the depth of information concerning particular places varies. Altogether, 77 completed copies of Questionnaire 1 (62 in Cieszyn and 15 in Kraków) and 423 completed copies of Questionnaire 2 (all in Cieszyn) containing information on edible plants were found. Several copies of Questionnaires 1 and 2, which had been mistakenly used, instead of Questionnaires 3 and 4, to record data on ethnomedicine, and records on collecting fungi (in 19 copies of Questionnaire 1) were discarded. Only 235 copies of Questionnaire 2 had herbarium specimens attached to them and many specimens were of bad quality, as they were collected by non-botanists (usually one shoot or one leaf, rarely flowers).

**Table 1:** English translation of Professor J. Gajek's Questionnaire form no. 2. The booklet containing a set of identical questionnaires began with a header containing the location and details of the researcher (detailed address, all places they had lived, occupation, level of education) and the informants (names, dates and places of birth, places of habitation before 1939, occupations).

No.	Question
1	Write the local name, commonly used by people.
2	Write other names, used more rarely in the locality.
3	Write names, ages and birth places of people who call this plant different names.
4	Specify the collection place (forest, arable field, meadow, scarp etc.) and date:
5	Mark months when the plant is collected for food with "x" [a table of months follows].
6	Write the folk names of the edible part of the plant.
7	Do the oldest people remember this plant being collected in the past? When?
8	Is it collected now?
9	If it is no longer collected, specify why.
10	Who collects the plant (children, the elderly, women, men?).
11	Is it only collected during spring food shortages or in times of famine (e.g. war?). Answer precisely!
12	Is this plant only collected and eaten by children or also by adults?
13	Specify the names of dishes made with this plant.
14	Do people store this plant for winter? How is it stored?
	Space for other remarks.
	Scientific name.

All the correspondents, whose details had been given in Questionnaire 2, were local, either living in the village (or town) which they wrote about (47 people) or in a nearby town (8 people). Most correspondents sent a set of questionnaires concerning one place only, apart from three people who supplied information for one or two more places. Most of those whose profession is known were teachers (22) and farmers (8), but at least three students, three priests, two officials, two lawyers, a group of scouts, a forester, a museum director and a director of a cultural centre also took part in the study. Hiking clubs for young people (*Koła Krajoznawcze Młodzieży Szkolnej*) from Kraków area also took part in the study supplying fifteen copies of Questionnaire 1. Apart from using their personal experience of living in the given place, the participants interviewed between one and six, usually elderly, people (mean number 2.5, modal value 2). It was not stated by the interviewers where the conversations with local people took place (indoors or outdoors) or if herbarium specimens were collected during the interviews or after. From context it can be presumed that both situations occurred.

The information contained in Questionnaires 1 and 2 (together with later data) has been, so far, used in three published maps of the Polish Ethnographic Atlas [2], i.e. in the map of the use of tree sap (no. 311), bread additives (no. 322) and *Vaccinium uliginosum *(no. 310). Twelve maps (no. 356–367) documenting the use of 15 taxa are still awaiting publication [3]. The Polish Ethnographic Atlas team was more interested in widely used species, the ways in which they were used and their local names and regional differences, than in tracking ethnobotanical curiosities used in a few villages. The number of questionnaires returned was not sufficient to construct detailed maps, which was the main objective of the Polish Ethnographic Atlas, so another study of wild edible plants was launched in 1964–69. This time it was done within a large project on all aspects of material culture, studied in a pre-selected grid of over 300 villages (Questionnaire 6). The questionnaire concerned was over a hundred pages long, which was the reason why it was often filled in hastily and superficially. No herbarium specimens were collected at that time [4,5].

Questionnaires 3 and 4, concerning the use of medicinal plants had already been used by Paluch [6] in his review of Polish ethnomedicine, but only information about the more commonly used species was published.

It seems that there was insufficient contact with botanists during the course of work on the Polish Ethnographic Atlas concerning ethnobotanical issues, as many herbarium specimens were incorrectly identified (sometimes even the genus being wrongly identified) and some botanical mistakes appeared in publications, e.g. confusion between *Chenopodium *and *Origanum*, due to their similar Polish names [4], or the assumption that all *oset *species belong to the scientific genus *oset *(*Carduus*), when the herbarium specimens and other studies clearly showed that most plants called *oset *belong to the genus *Cirsium *[4]. These few mistakes, however, do not diminish the great effort put into the documentation of the use of plants, and the professionalism shown in constructing the questions, especially for Questionnaire 2 (Table 1).

Summing up, why is this set of documents so important to ethnobotany?

1. It was collected in 1948, just after World War II, when the memory of famine plants was fresh and poverty preserved gathering traditions.

2. It is one of the earliest examples of a purely ethnobotanical herbarium in Europe.

3. It uses free lists of plants; which constitute a very valuable resource for ethnobotanical research [7,8], as no species or mode of use had been pre-suggested.

The aim of this study is to present the content of Questionnaires 1 and 2, with particular attention paid to:

1. rarer species, whose use had not previously been reported in Poland.

2. assigning specific scientific names to folk taxa, previously referred to in ethnobotanical literature by only, or mainly, folk generic names (e.g. *rdest, mięta, oset, mlecz, szczaw, ślaz*)

3. finding places with a high incidence of the use of wild edible plants.

**Figure 1:**
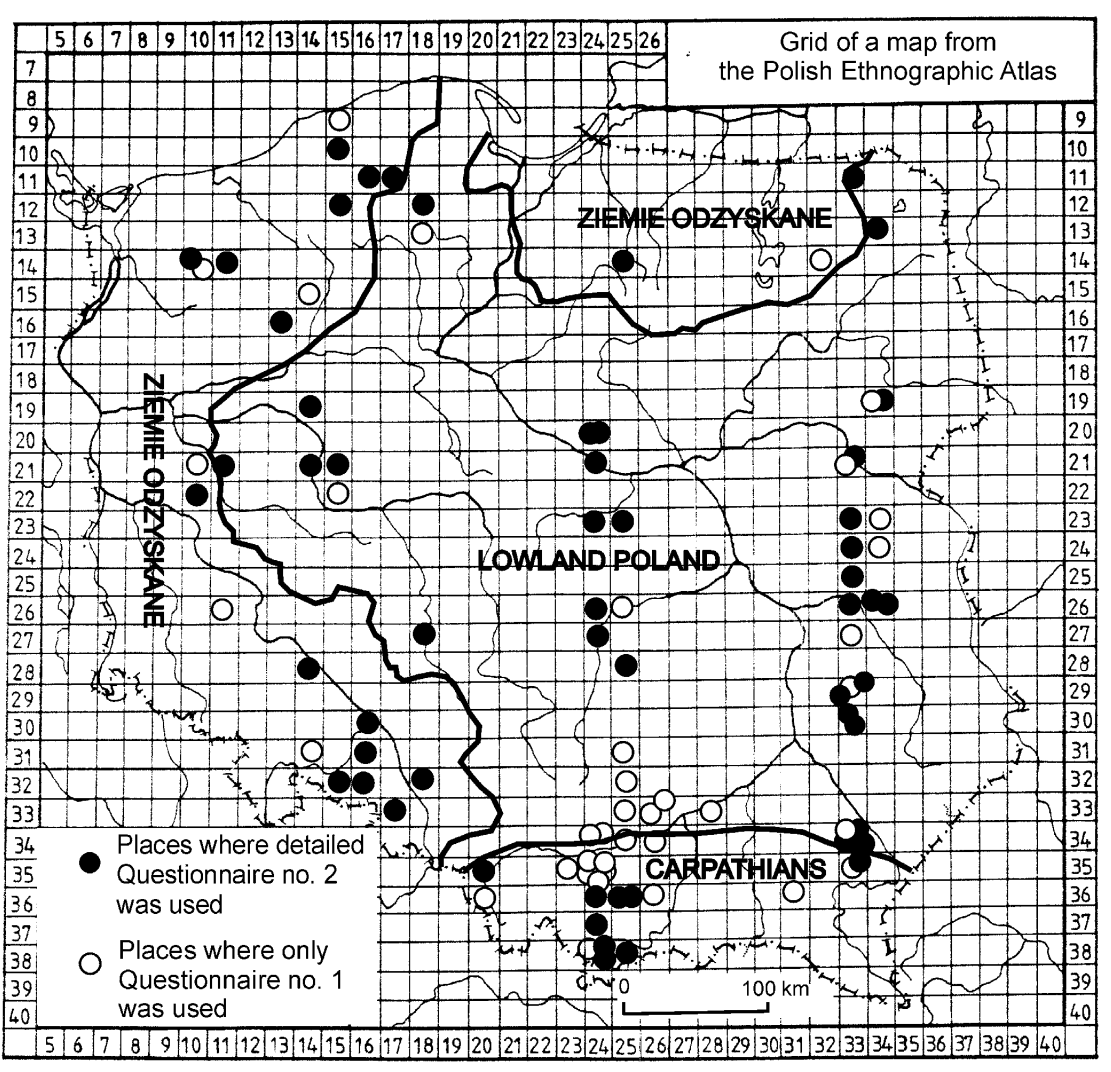
Distribution of the studied localities.

The geographical and historical background of the use of edible plants in Poland was sketched in an earlier study [1]. The most important point for a reader of this study is that, in terms of ethnography and rural culture, Poland can be divided into three zones (Fig. 1):

• the Carpathians, a conservative traditional area, subject to the largest amount of ethnographic studies,

• the western and northern outskirts of Poland (*Ziemie Odzyskane*, i.e. *Reclaimed Lands*), largely ignored by ethnography, as they were reclaimed from Germany after World War II, and are inhabited mainly by Poles moved from the Soviet Union after WWII, with a small scattering of Poles and Germans who lived there before 1939 (most Germans or people of mixed origin left for Germany),

• the rest of Poland (lowland Poland), where rural culture has been studied in the most interesting regions, but on the whole less intensely than in the Carpathians.

## Methods

A database was created to analyse the content of the questionnaires. When a species from the same locality occurred in both Questionnaire 1 and Questionnaire 2 (which was usually the case), the presence of the species was calculated only once. Exactly 1000 (species × locality) records occur in the database. All herbarium specimens were examined and the taxa which were harder to identify were cross-checked with the plant taxonomist, Dr Krzysztof Oklejewicz (Rzeszów University).

Folk names used in the questionnaires often refer to the whole genus. Particular scientific species names were assigned to them when:

1. they represented a monospecific genus in a given region (distributions were checked with Zając and Zając's Atlas [9]),

2. other species of the genus were extremely rare in Poland,

3. all the herbarium specimens for the genus were identified as the species in question, and the field experience of the author suggested that the records unsupported by herbarium specimens contain the same species.

Latin names of plants are listed according to Flora Europaea [10], and main synonyms are given, including the name in the current checklist of Polish vascular plants of Poland [11] and older names used in the analysed materials. All local names occurring in the questionnaires are given as well.

Lists of edible plant species were made for 95 different places (Fig. 1, Table 2), nine of these lists, however, included only one or two species and were excluded from statistical analysis, as they contained obviously superficially collected material. The remaining 86 localities, each with a list containing three or more species, were divided for the purpose of analysis into three main regions: the Carpathians, yielding 19 localities, Ziemie Odzyskane – 20 and the rest ('lowland Poland') – 47.

**Table 2:** Characteristics of the studied localities (ordered from north to south).

Place	District (*Powiat)*	R	A	P	S	SN	Q1	Q2	I
Możdżanowo	Słupsk	Pm	Z	9	15	5	1	0	nd
Barwino	Słupsk	Pm	Z	10	15	39	1	20	3
Tuchomie	Bytów	Pm	Z	11	16	14	1	6	1
Studzienice	Bytów	Pm	Z	11	17	11	1	6	1(1)
Przerośl	Suwałki	Ps	L	11	33	30	1	10	5
Słosinko i Falkowice	Bytów	Pm	Z	12	15	12	1	6	3(3)
Borsk	Kościerzyna	Pm	L	12	18	4	1	2	2
Wiele	Kościerzyna	Pm	L	13	18	4	1	0	nd
Bargłów Dworny	Augustów	Ps	L	13	34	1	0	1	3
Radowo Małe	Łobez	Zp	Z	14	10	23	1	0	nd
Sielsko	Łobez	Zp	Z	14	10	2	0	1	2
Wętno	Drawsko	Zp	Z	14	11	5	0	5	nd
Biesal	Olsztyn	Wm	Z	14	25	22	1	11	2(1)
Jagodzin	Pisz	Wm	Z	14	31	1	two letters	0	nd
Jastrowie	Wałcz	Wp	Z	15	14	5	1	0	nd
Wałcz	Wałcz	Zp	Z	16	13	4	0	4	5
Oborniki	Oborniki	Wp	L	19	14	9	1	9	4
Kalnica	Bielsk Podlaski	Ps	L	19	34	13	1	8	3
Oleksin	Bielsk Podlaski	Ps	L	19	34	18	1	0	nd
Biała – Stara	Płock	Mz	L	20	24	5	1	4	2
Płock	Płock	Mz	L	20	24	10	0	10	1
Brójce	Międzyrzecz	Lb	Z	21	10	4	1	0	nd
Wąsowo	Nowy Tomyśl	Wp	L	21	11	4	1	4	3
Kobylniki Zdrój	Poznań	Wp	L	21	14	14	1	10	2
Szreniawa	Poznań	Wp	L	21	15	7	0	7	nd
Pepłowo	Płock	Mz	L	21	24	6	1	8	nd
Korczew	Siedlce	Mz	L	21	33	21	1	0	nd
Szczeglacin	Siedlce	Mz	L	21	33	14	1	14	1
Nowe Kramsko	Zielona Góra	Lb	Z	22	10	7	0	7	4
Kórnik	Poznań	Wp	L	22	15	11	1	0	nd
Lisiewice	Łowicz	Łd	L	23	24	16	1	10	2
Płaskocin	Łowicz	Łd	L	23	25	8	1	8	2
Radomyśl	Siedlce	Mz	L	23	33	10	0	10	2
Grochówka	Siedlce	Mz	L	23	34	26	1	0	nd
Suleje	Łuków	Lu	L	24	33	9	0	9	2
Drelów	Biała Podlaska	Lu	L	24	34	19	1	0	nd
Wola Osowińska	Radzyń Podl.	Lu	L	25	33	31	1	9	3 plus children
Lubin Legnicki	Lubin	Ds	Z	26	11	3	letter	0	nd
Zamość	Piotrków Trybunalski	Łd	L	26	24	12	1	10	3
Glinnik	Tomaszów Maz.	Łd	L	26	25	5	1	0	nd
Białobrzegi	Lubartów	Lu	L	26	33	1	1	1	4
Berejów	Lubartów	Lu	L	26	34	4	1	4	nd
BrzeŁnica Bychawska	Lubartów	Lu	L	26	34	14	1	14	1
Świba	Kępno	Wp	L	27	18	4	0	4	3
Zarzęcin	Opoczno	Łd	L	27	24	10	1	10	3
Nasutów	Lublin	Lu	L	27	33	23	1	0	nd
Wrocław – Swojec	Wrocław	Ds	Z	28	14	8	1	16	2(1)
Marcinków – Władysławów	Opoczno	Łd	L	28	25	7	1	7	4
Wilkołaz	Kraśnik	Lu	L	29	33	16	1	0	nd
Wólka Rudnicka	Kraśnik	Lu	L	29	33	6	1	6	nd
Dębszczyzna	Lublin	Lu	L	29	33	10	0	10	1
Oldrzyszowice	Brzeg	Op	Z	30	16	5	1	5	1
Huta Józefów	Kraśnik	Lu	L	30	33	3	0	4	nd
Polichna	Kraśnik	Lu	L	30	33	20	1	9	nd
Wigończyce	Ząbkowice Śl.	Ds	Z	31	14	1	1	0	nd
Bielice	Nysa	Op	Z	31	16	9	1	9	1
Deszno	Jędrzejów	Sw	L	31	25	11	1	0	nd
Dębie	Opole	Op	Z	32	15	4	1	4	1
Rzymkowice	Nysa	Op	Z	32	16	7	1	5	2
Większyce	Kedzierzyn Koźle	Op	Z	32	18	10	0	10	2
Janowice	Złotów	Mp	L	32	25	12	1	0	nd
Maciowakrze	Kędzierzyn-Koźle	Op	Z	33	17	4	0	4	1
Krasieniec Zakupny	Kraków	Mp	L	33	25	13	1	0	nd
Gruszów	Proszowice	Mp	L	33	26	6	1	0	nd
Dalechowice	Kazimierza Wk	Sw	L	33	26	11	1	0	nd
Wielopole	Dąbrowa Tarnowska	Mp	L	33	28	7	1	0	nd
Kraków – Bronowice Wielkie	Kraków	Mp	L	34	24	3	1	0	nd
Michałowice	Kraków	Mp	L	34	24	16	1	0	nd
Szarocie near Brzezie	Wieliczka	Mp	C	34	25	8	1	0	nd
Dziewin	Bochnia	Mp	L	34	26	7	1	0	nd
Ruda Łańcucka	Łańcut	Pk	L	34	33	1	0	1	6
Sonina	Łańcut	Pk	L	34	33	3	1	3	1
Laszczyny	Leżajsk	Pk	L	34	33	10	1	10	3
Grzęska	Przeworsk	Pk	L	34	33	12	1	0	nd
Pruchna	Cieszyn	Sl	C	35	20	18	2	18	2
Brzeźnica [only name of local post office]	Wadowice	Mp	C	35	23	18	1	0	nd
Kraków – Sidzina	Kraków	Mp	C	35	24	4	1	0	nd
Mogilany	Kraków	Mp	C	35	24	2	1	0	nd
Włosań	Kraków	Mp	C	35	24	2	1	0	nd
Zelczyna	Kraków	Mp	C	35	24	14	1	0	nd
Krzywaczka	Myślenice	Mp	C	35	24	3	1	0	nd
Pantalowice	Przeworsk	Pk	C	35	33	7	1	7	1
Sietesz	Przeworsk	Pk	C	35	33	10	1	0	nd
Skoczów	Cieszyn	Sl	C	36	20	28	1	0	nd
Skomielna Czarna	Myślenice	Mp	C	36	24	10	letter	10	1
Mszana Dolna	Limanowa	Mp	C	36	25	6	1	4	6
Szczyrzyc	Limanowa	Mp	C	36	25	18	1	10	5
Limanowa	Limanowa	Mp	C	36	26	10	1	0	nd
Trześniów	Brzozów	Pk	C	36	31	8	1	0	nd
Rabka Zdrój	Nowy Targ	Mp	C	37	24	14	0	14	5
Szaflary	Nowy Targ	Mp	C	38	24	28	1	0	nd
Biały Dunajec – Stołowe	Zakopane	Mp	C	38	24	7	1	7	3
Poronin	Zakopane	Mp	C	38	24	5	2	5	1
Frydman	Nowy Targ	Mp	C	38	25	21	1	3	2
Boża Wola	exact location imprecise (11 villages with this name)	Mz?	L	?	?	1	short note	0	nd

R – region (*województwo*, abbreviations of region names see Appendix); A – area according to the author's division into Z, L and C, where Z = Ziemie Odzyskane (areas reclaimed from Germany, where most population post-1945 are immigrants from the expanded Soviet Union, L = lowland Poland, C = the Carpathians; P – latitudinal coordinates on the grid of the Polish Ethnographic Atlas map (Fig. 1), numbers increasing from north to south; S – longitudinal coordinates on the grid of the Polish Ethnographic Atlas map (Fig. 1), numbers increasing from west to east; SN – species number per locality; Q1 – number of copies of Questionnaire 1; Q2 – number of copies of Questionnaire 2; I – number of informers (when available), if indigenous inhabitants and immigrants were distinguished, the number of the former was given in parentheses; nd – no data.

There has been a strong emphasis in recent ethnobotanical literature on the quantification of results and the elimination of the publication of accidental findings based on information gathered from single individuals. Although each of the correspondents who sent questionnaires to the Polish Ethnographic Atlas sent information recorded from only one or a few selected people, the use of most species was documented from at least a few different locations, where data were collected by different researchers. Although reports of the use of species from one locality may easily be false, e.g. due to the inclusion of a mistaken herbarium specimen, I have provided exact locations of these species in order to enable verification of the information in the relevant locations with the local population, who may still remember the name of the folk taxa, even if the former use of plants has been forgotten.

## Results

### General characteristics

Ninety-eight taxa of wild edible plants were identified in Questionnaire 1 and Questionnaire 2, seventy-one of these species were confirmed by herbarium specimens (see Appendix). The category of fruits and seeds was the most highly represented (35 species and 45% of all the *species × locality *records). Green vegetables (any species whose leaves, shoots or unripe fruits are consumed in quantities larger than for flavouring) were represented by 31 species (26% of *species × locality *records), beverages (infusions, sap and wine) by 26 species (9% records), flowers eaten for their sweet taste by 8 species (2%), spices/flavourings (any parts of plants) – 6 species (5%), and underground parts used for food – 6 species (3%).

The most commonly listed plants were two green vegetables: *Rumex *spp., appearing in 75 localities, and *Chenopodium album*, in 50 (Table 3). Next in the ranking were three taxa bearing fleshy fruits: *Rubus *subgenus *Rubus *('blackberries'), *Fragaria vesca *and *Vaccinium myrtillus*, followed by another green vegetable, *Urtica *spp., two species bearing fruits: *Vaccinium vitis-idaea *and *Rubus idaeus *and then a children's snack, *Oxalis *spp. (Table 3).

**Table 3:** Frequency list of the thirty-two most commonly collected taxa. The list includes sixteen records of *głóg*, a term which could refer to either *Crataegus *or *Rosa *(both taxa are equally frequently called by this name), which were assigned in the proportion of 8:8 to each of the genera.

Species	Number of localities	Parts and main modes of use
*Rumex *spp.	75	leaves, raw and cooked in soups
*Chenopodium album*	50	leaves, boiled and fried, mainly as famine food
*Rubus *subgenus *Rubus *spp.	48	fruits, raw and in preserves
*Fragaria vesca*	47	fruits, raw and in preserves
*Vaccinium myrtillus*	47	fruits, raw and in preserves
*Urtica *spp.	38	leaves, boiled and fried, mainly as famine food
*Vaccinium vitis-idaea*	34	fruits, raw and in preserves
*Rubus idaeus*	32	fruits, raw and in preserves
*Oxalis *spp.	31	leaves, raw, as children's snack
*Corylus avellana*	29	fruits, mainly raw
*Rosa *spp.	29	fruits, raw and in preserves
*Prunus spinosa*	27	fruits, raw and in preserves, largely as children's snack
*Armoracia rusticana*	26	roots, grated, as meat condiment
*Betula *spp.	26	sap, mainly as children's beverage
*Sambucus nigra*	24	fruits, preserved
*Crataegus *spp.	22	fruits, raw and in preserves, largely as children's snack
*Carum carvi*	19	seeds, added to bread and sauerkraut
*Elymus repens*	19	rhizomes, dried and ground, used only as famine food
*Tilia *spp.	17	flowers, infusion
*Acorus calamus*	13	young shoots, only as children's snack
*Trifolium *spp.	13	nectar from flowers sucked by children
*Centaurea cyanus*	12	petals used by children to make a fermented drink with sugar
*Quercus robur*	12	acorns, roasted to make coffee
*Mentha longifolia & M. arvensis*	11	infusion, soups and dumplings
*Prunus padus*	11	fruits, raw as children's snack
*Ribes nigrum *&*R. spicatum*	11	fruits, raw or preserved covered in sugar or as juice and jam; only in eastern and northern Poland
*Taraxacum *spp.	10	leaves, raw, served like lettuce
*Vaccinium uliginosum*	10	fruits, raw or as juice, jam, soup or compote;
*Fagus sylvatica*	9	raw nuts eaten by children
*Malva sylvestris & M. neglecta*	9	immature fruits, eaten raw, mainly in western Poland
*Sorbus aucuparia*	9	fruits, juice, jam, soup, liqueurs
*Vaccinium oxycoccos*	9	fruit, usually in jams and sauces

The sour-tasting leaves and shoots of *Rumex *spp. were commonly collected throughout the country to make soup. They were sometimes stored for winter, pressed tightly with salt in sealed bottles. They were also the commonest children's snack. *Chenopodium album*, *Urtica dioica *and *U. urens *were described, in most questionnaires, as poor people's or famine food used only until the beginning of the 20^th ^century or during World War Two. Still used in several localities at the time of study, they were, however, associated with old-fashioned habits or poverty and collected mainly by elderly women. *Chenopodium *leaves were usually briefly boiled, drained, fried and then garnished with cream or milk. *Urtica *leaves were used in a similar way (often together with the former) but they tended to be used in soups.

The fruits used in at least a quarter of the studied localities were: *Rubus *subgebus *Rubus, Rubus idaeus, Vaccinium myrtillus, V. vitis-idaea, Fragaria vesca, Corylus avellana, Rosa *spp., *Prunus spinosa *and *Sambucus nigra*. The following fruits were used in fewer localities, but their use must still have been widespread throughout the country (they were recorded in at least nine localities): *Crataegus *spp., *Prunus padus*, *Ribes *spp., *Vaccinium uliginosum*, *V. oxycoccos, Fagus sylvatica *and *Sorbus aucuparia*. Most wild fruits were collected by women and/or children (Table 4), eaten raw or made into juice, jam or wine. Fruits were often stored for winter with added sugar (informers often complained about the high price of sugar, particularly when describing the use of *Vaccinium vitis-idaea*). *Vaccinium myrtillus *fruits were also dried. *Corylus, Fagus *and *Prunus padus *were mainly eaten raw.

**Table 4:** Specialization in the collecting of wild edible plants.

	%						
		
	Everyone	Women and children	Only children	Only women	Only adults	Men and children	NUMBER OF ANSWERS
*Vaccinium myrtillus*	55	36	9				22
*Rubus idaeus*	54	31	15				13
*Fragaria vesca*	48	39	13				23
*Armoracia rusticana*	45	18	9	18	9		11
*Vaccinium vitis-idaea*	38	50	13				16
*Rubus *subgenus *Rubus*	30	35	35				23
*Corylus avellana*	29	7	36			29	14
*Rumex *spp.	23	45	30	3			40
*Prunus spinosa*	20	10	60	10			10
*Chenopodium album*	18	36	9	36			22
*Urtica *spp.	18	23		45	14		22
*Oxalis *spp.	8		92				13
*MEAN*	31	31	24	10	2	2	229

A very limited range of wild plants was used as spices and flavouring. *Carum carvi *seeds were used to flavour bread and sauerkraut, and grated *Armoracia rusticana *roots were added to meat, boiled beetroot or other salads. In the southern and eastern part of Poland mint leaves (*Mentha *spp.) were used to flavour dumpling filling or soups.

Apart from foods, the questionnaires contain information on herbal infusions drunk by healthy people on an everyday basis, these included two taxa used commonly throughout the country: infusions of *Tilia cordata *flowers and roasted *Quercus robur *acorns (use of the latter was nearly obsolete in 1948), and taxa used rarely and locally on an everyday basis (used more often medicinally): *Rubus *subgenus *Rubus, Rubus idaeus *and *Fragaria vesca *leaves, *Mentha arvensis, Chamomilla recutita, Galium odoratum, Viola tricolor, Thymus pulegioides *and *Thymus serpyllum *flowering shoots. In Maciowakrze, near Opole, *Rumex obtusifolius *leaves were added to compotes.

The studied questionnaires contain information on the use of species not listed in the review of edible plants of Poland [1]. These are: *Rumex thyrsiflorus *(leaves), *R. obtusifolius *(leaves), *Ribes spicatum *(fruits), *Cirsium arvense *(leaves), *Lamium album *(flowers and leaves), *Stratiotes aloides *(whole plants), *Galium odoratum *(flowering shoots), *Sedum maximum *(underground parts), *Parthenocissus *sp. (fruits), *Stachys palustris *(rhizomes), *Tragopogon pratensis *(shoots), *Viola hirta *(roots), *V. tricolor *(flowering shoots for tea), *Viscum album *(fruits), which were confirmed by herbarium specimens, whereas *Chamomilla recutita *(flowering shoots for tea), *Papaver rhoeas *(seeds), *Primula elatior *(flowers) and *Ulmus *sp. (fruits and bark) were identified on the basis of folk names and additional information in the questionnaires.

### Children as main collectors of wild plants

According to the results of this study it was mainly women and children who collected wild plants. For the 12 most commonly collected taxa, the following categories scored around 30% in question number 10 (*who collects the plants*): 'children and women' (32%), 'everyone' (32%) and 'only children' (24%) (Table 4). When we look at particular species the above mentioned ratio is usually similar, with a few exceptions: *Oxalis *spp. collected mainly by children, *Corylus avellana *collected mainly by men and children, and *Urtica *spp. which were not collected by children, but by adults, mainly women (Table 4).

When rarer taxa are included, the proportion of answers 'collected only by children' is similar (21%, 81 out of 389). It must be stressed however that although children were the most important plant collectors, they also collected plants for the adults, as the proportion of answers 'eaten only by children' is lower, at only 5% (20 out of 389).

The commonest children's snack, *Oxalis *spp., ranked just between such important wild crops as *Rubus idaeus *and *Corylus avellana*. Other commonly collected species, which were eaten mainly by children were *Prunus spinosa, Crataegus *spp. and *Rosa *spp. (these three taxa were, to a lesser extent, also used to make preserves). Other species of predominantly children's snacks included: young shoots of *Acorus calamus*, a few species of flowers, whose nectar was sucked, particularly *Trifolium pratense, T. repens, Robinia pseudoacacia, Lamium album, Symphytum officinale*, fruits of *Prunus padus, Rubus saxatilis, Maianthemum bifolium, Frangula alnus, Malva *spp., *Capsella bursa-pastoris *and the sweet rhizomes of *Polypodium vulgare*. Surprisingly, in a country where children have always been discouraged from drinking alcohol, several respondents wrote about children independently making a kind of "wine", particularly with the petals of *Centaurea cyanus*, which were fermented for some time with water and sugar (12 reports from various regions). The inventory of children's snacks seems to be uniform across the country with very few regional differences.

### Geographic variation

**Figure 2:**
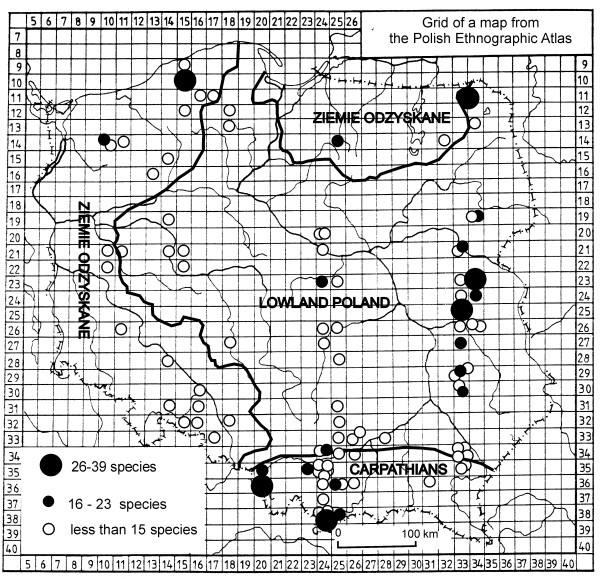
Localities with the longest lists of edible plant species in 1948.

The mean number of species used in a single locality (calculated from lists longer than 2 species) was 11.3, the modal value was 10. There was high variation in the number of species used per locality (SD = 7.5). The longest list, 39 species, was recorded in Barwino, near Słupsk (Pm). This village used to be a part of Germany before 1939, so most of the inhabitants were removed after World War II, and the list was based on interviews with four newcomers from the area of Łuck (present Belarus). The second longest list, 31 species, was recorded in eastern Poland, in Wola Osowińska, near Łuków. Lists ranging from 20 to 30 species were recorded in localities from a few regions of Poland (mainly E and S, but also NE and NW), with the exceptions of central, western and south-western Poland (Fig. 2).

The mean number of species per locality was highest in the Carpathians (12.5 ± 1.7 SE), lower in lowland Poland (11.4 ± 1.0 SE) and lowest in Ziemie Odzyskane, the areas reclaimed from Germany (10.4 ± 2.1 SE). The difference was not significant between any of the pairs of the three above mentioned parts of Poland (Mann Whitney U test, P > 0.05) Differences were larger if we look at modal values, which were 10 both for the Carpathians and lowland Poland and only 7 for Ziemie Odzyskane, since the mean species number per locality for this area was elevated by a few very species-rich lists.

There was no correlation between latitude and the number of species listed (Spearman rank correlation coefficient, rho = 0.01, P = 0.89), however there was a small but significant correlation between the longitude and the species number (Spearman rank correlation coefficient, rho = 0.29, P = 0.007). Almost identical results were obtained when localities from Ziemie Odzyskane, dominated by immigrant populations, were not included in the calculations, although, in this case, neither of the correlations were significant (rho = -0.004, P = 0.98 for latitude and rho = 0.23, P = 0.07 for longitude).

**Figure 3:**
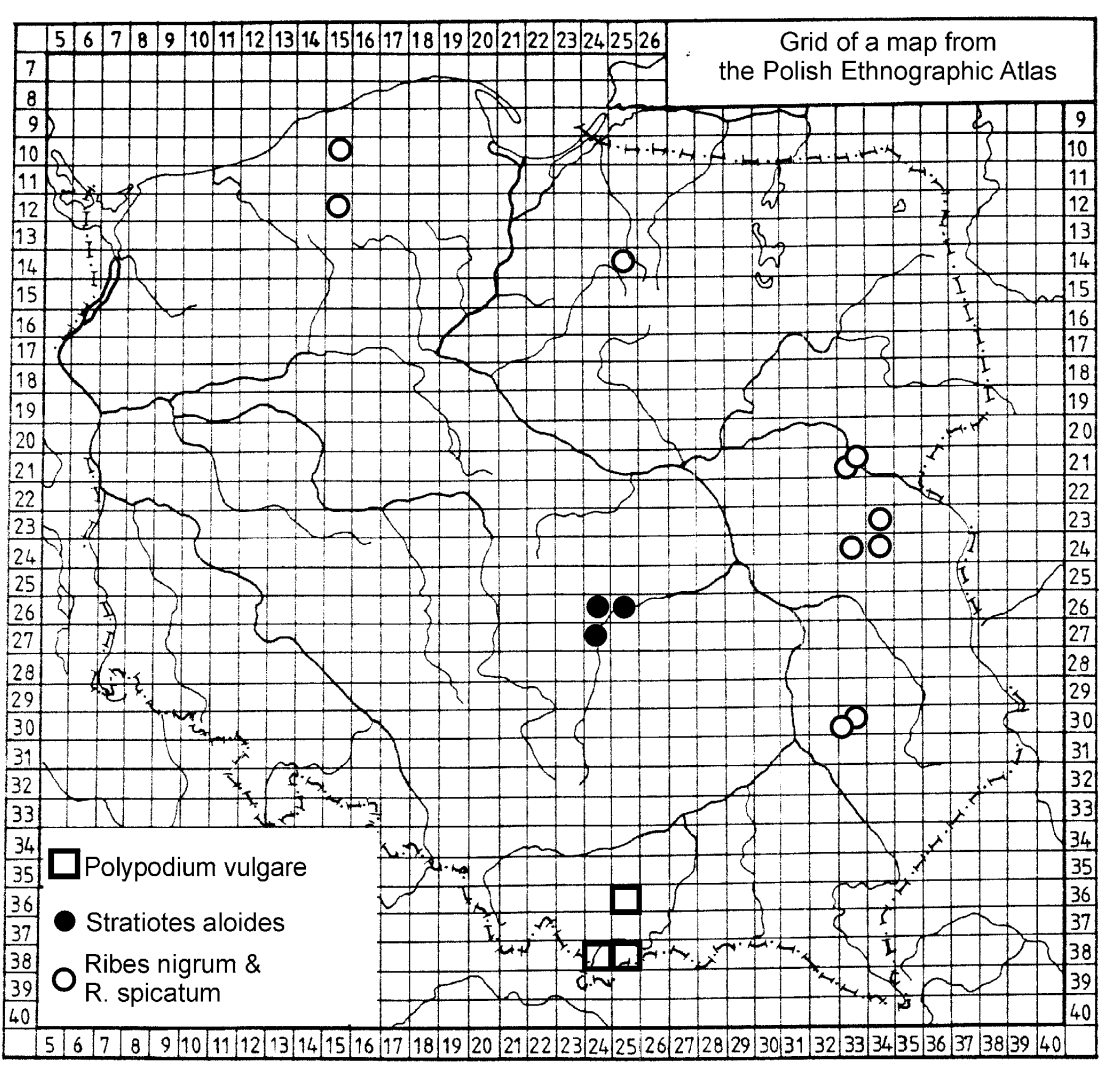
Distributions of the use of species showing strongly regionalised patterns.

The use of more common species did not show any strong geographical patterns. The use of some species recorded rarely (1–3 localities) was probably more regionalised. A strong regional pattern can be noticed only in the distribution of a few species. *Ribes *fruits were gathered from the wild only in eastern and northern Poland, *Polypodium vulgare *rhizomes were used only in the Carpathians, *Stratiotes aloides *was used only in central Poland (Fig. 3) and *Malva *spp. were used predominantly in western Poland.

## Discussion

### Ratio of botanical species in folk taxa

The herbarium specimens made available help to answer questions which were raised by Łuczaj & Szymański's review [1]: they reveal the exact proportions of particular species contained in folk taxa, which were impossible to estimate from descriptive ethnographic works.

Within the folk taxon *szczaw *the proportion of *Rumex acetosa, R. acetosella *and *R. thyrsiflorus *is 16:4:1. The latter species has never been mentioned in ethnobotanical literature before. It is easily confused with *R. acetosa*, it is equally large, however it flowers later and grows in dry, sandy soils where *R. acetosa *is not found.

Some publications [1,12] suggest that within the folk taxon *lebioda/łoboda *a variety of *Chenopodium *and *Atriplex *species were used. However all 16 herbarium specimens from this taxon belong to *Chenopodium album*.

The herbarium specimens confirm that both nettle species (*Urtica dioica *and *U. urens*) were used (ratio 9:4).

All previous ethnographic publications put an equation mark between *szczaw zajęczy *and *Oxalis acetosella*, however specimens of *Oxalis stricta *s.l. were also found in the analyzed material (ratio 9:2). *O. stricta *is an alien species, which occurs mainly on arable land, so it may have been, in some villages, more available to children than the woodland species, *O. acetosella*.

Most mint specimens were unidentifiable, however both *Mentha longifolia *and *M. arvensis *were found in the material. Their use had been reported before [1], but only in single locations. The majority of wild mints used as cooking herbs or for infusions must have belonged to these two species.

The use of both species of birch, *Betula pendula *and *B. pubescens *was confirmed (ratio 4:1).

*Crataegus *and *Rosa *are difficult to distinguish in ethnographic materials, as they are often called *głóg*, and were used in a similar fashion. All the identified herbarium specimens from these genera belong to *Crataegus monogyna *(6), whereas the five specimens of *Rosa *are probably *R. canina*, but full identification is impossible due to the lack of fruits.

All the specimens called *mlecz *(the scientific name for *Sonchus*) belong to the genus *Taraxacum*, and not *Sonchus*, which creates a suspicion that previous reports on the use of *Sonchus *are botanical mistakes.

Both *Malva sylvestris *and *M. neglecta *were called the same folk names, the ratio of their specimens is 3:1.

Due to the lack of flowers and fruits in the preserved *Rubus *specimens it was not possible to identify particular species, except for *Rubus caesius*, which constitutes a surprisingly large proportion of specimens (ratio 6:7). Its popularity as a food plant, although its berries are some of the smallest and sourest of the blackberry species, can be attributed to the fact that in many areas of Poland (especially NE) other *Rubus *subgenus *Rubus *species are rare [13].

### Little known edible species

Out of the newly recorded species, probably the most valuable finding is the discovery of the use of *Stratiotes aloides*. This water plant, although it occurs over a large area of Poland, was an important famine plant only in the Łódź area (Glinnik, Zamość and Zarzęcin), where, until the turn of the 19^th ^and 20^th ^century, it was commonly collected from the bottom of lakes and cooked.

In Mszana Dolna (W Carpathians) *Viola hirta *roots were eaten as a children's snack, and called *słodkie korzenie *('sweet roots'). Such properties of *V. hirta *have never been reported before, and as this information is based on one herbarium specimen, it needs further confirmation.

*Stachys palustris*, recorded in the village of Biały Dunajec – Stołowe (W Carpathians) is another famine plant previously under-recorded by Polish ethnographers. It was not listed in Łuczaj and Szymański's review [1], it was, however, recorded by Rostafiński [14] in 1888.

The studied questionnaires also contain information on a third locality in the Western Carpathians (Rabka) where *Heracleum sphondylium *was used to make soup, until the 20^th ^century.

A few of the presented plants have not been considered as edible in Europe, but were consumed in other parts of the world: related *Maianthemum *species fruits and *Oxalis stricta *leaves by Native Americans [15], *Convolvulus arvensis *s.l. shoots and *Ulmus *fruits by the Chinese [16] and *Viscum *fruits in Nepal [17].

### Unidentified species needing verification

In a few dozen records neither scientific species or genus name could be matched, sometimes due to the fact that a folk name is commonly used to describe two genera, e.g. 'babka' for *Plantago *and *Malva *and 'głóg' for *Crataegus *and *Rosa*. The use of the seeds of a plant called *anyżek *(literally 'little anis') was reported in a few places. This may be *Pimpinella saxifraga *or some other aromatic *Apiaceae *plant. Near Nowy Targ (Mp) the use of some underground bulbs called *orzechy ziemne *('earth nuts') in Szaflary and *ziemne jabłka *('earth apples') in Frydman was recorded. This is most likely *Lathyrus tuberosus*, which was earlier recorded in SE Poland in the Rożnów area [18] or *Helianthus tuberosus*. In Zelczyna near Kraków, *koniczyna wodna *('water clover') was eaten. This folk name may refer to either *Menyanthes trifoliata *or *Marsilea quadrifolia*.

### Children – an important vector of ethnobotanical knowledge

It is practically a cliché that in hunter-gatherer societies it was mainly women who gathered food, while men hunted [19]. As far as the children's contribution to subsistence effort is concerned, it varies. In some hunter-gatherer societies, such as Hadza, children's participation is important, whereas among !Kung they contribute little to gathering [20,21]. In the mid-20th century Polish countryside, where both men and women were strongly involved in farming practices, children, outside school hours, served as shepherds, and were the group in society which had the most contact with wild plants, often even replacing women as the main gatherers. The transmission of traditional ethnobotanical knowledge in such circumstances is an interesting issue. We can presume that traditional knowledge transfer was horizontal, with older children showing new plants to the younger and this conclusion can be supported by the authors' experiences from field interviews with older people. As children usually ate plants raw, this horizontal transfer mainly concerned plant recognition, whereas traditional knowledge on the preparation of cooked and fried dishes (jams, juices, soups) was probably passed vertically from mothers to daughters, as in many traditional societies [22]. Such a mixture of horizontal and vertical transfer of knowledge has been well documented recently in Thailand [23].

A large proportion of plants, eaten around 1948 mainly as children's snacks, are probably forgotten articles of adult food, e.g. *Oxalis *leaves, which were used to make soup, and *Trifolium *flowers, which were eaten as famine food [1]. Thus children's interest in snacking on wild plants had an adaptive value: these plants could be used in larger quantities in times of food shortages. The repertoire of children's snacks constituted a reserve list of edible plants for the community. Sometimes they were just plants which did not occur in large quantities or were time-consuming to collect (e.g. *Equisetum arvense *and *Lathyrus tuberosus *bulbils, *Oxalis *leaves, *Polypodium *rhizomes) but could be used in emergencies or if they became more abundant. The part of plants used by children may differ from the most nutritious part (e.g. *Malva *fruits used by children versus leaves cooked by adults, *Lamium album *flowers versus leaves) but the notion that a given species was edible was preserved.

The knowledge that certain plants are edible and tasty, even if they remain exclusively a children's snack and no famine occurs for decades, could probably have been maintained through a few generations in traditional rural communities. Children spent a lot of time outdoors, served as shepherds and helped parents in collecting wild plants. Trying different tastes must have been exciting for people raised on the bland staples of potatoes, cereals and dairy. However within the last two decades since the fall of Communism in 1989, a larger drop in the knowledge of wild edible snacks has occurred among Polish children, according to the author's preliminary observations. This process can be easily explained by migrations from rural areas, the growing choice of food articles available (e.g. exotic fruits) and the increasing length of time spent by children indoors (e.g. when watching television or using computers).

### Comparison with other countries

Assuming that the lists in particular localities come close to representing the total traditional knowledge of a village community, they can be compared with parallel studies from other countries. In one area in Italy with over seven thousand inhabitants, 44 species are known [24] and in another village of southern Italy, Castelmezzano, with less than a thousand inhabitants, the use of 60 species of edible plants was recorded [25]. In a small traditional community of Mapuche Indians in temperate parts of Argentina (with climate similar to this of Poland), 24 wild edible plants species are known [26]. The results of this study, although they extend the list of edible plants of Poland considerably, still document relatively low levels of traditional knowledge in 1948, as on average only 11 species of plants (mainly common edible fruits) were listed, with the longest list consisting of 39 species. This avoidance of wild plants in the Polish diet, except for fruits, was extensively discussed in a previous article, where it was attributed mainly to cultural factors [1]. This study supports the cultural hypothesis (rather than putting blame on past researchers' neglect), as it is fruits and not green vegetables that make up the largest category, in sharp contrast to southern European countries, e.g. Spain [27], Bosnia and Herzegovina [28], or the central and southern part of Italy [29,30]. Poland is more similar to northern Italy, where the eating of bitter green vegetables is not popular [29]. Moreover the recorded use of spices and cooking herbs was extremely limited (5% of records) and incomparably lower than in southern Europe. The modern gathering habits analysed in another study [31] just a few years ago show a drastic reduction of wild food plants collected, particularly the non-fruit component, when compared to the results of the study carried out in the 1960s [31] and the results presented here. There seems to be a strong pattern of avoidance of using the green parts of plants, particularly strong tasting ones, as either vegetables or flavouring, during periods when food is not scarce. It is a matter of discussion whether this pattern could be referred to as 'herbophobia', a term analogous to Wasson's 'mycophobia' which commonly refers to the almost total absence of fungi in traditional English and German cuisine [32,33]. Although the use of green vegetables constituted 26% of all records, half of them concerned plants characterised by respondents as famine or poverty food and three quarters of all the records for green vegetables were made up by only four genera (*Rumex, Chenopodium, Urtica *and *Oxalis*). Among the twenty most frequently used taxa (Table 3), there are only five taxa whose green parts were used: two famine vegetables, two raw children's snacks and only one taxon – *Rumex*, perceived as a normal, non-famine cooked vegetable. In contrast to this, in the list of twenty culturally most significant wild plants in Garfagnana, Italy, even a few decades after 1948, as many as seventeen taxa were green vegetables or aromatic herbs. The term 'herbophilia' could apply to such cultures as that of Garfagnana and other parts of central and southern Italy, as well as China and Japan, in which the green parts of plants of numerous species are often used and highly prized [16,29,34]. On the other hand the term 'herbophobia' may not be fully adequate as it implies a phobia (an irrational, intense, persistent fear of something), whereas the phenomenon described is more of a disappearance of wild green vegetables from the diet, linked to their low cultural significance and associations with poverty (but not the devil, as was the case in some countries for mushrooms [33]). So maybe a term 'culinary a-herbia' (or 'aherbia') would be more appropriate for cultures which display no interest in using larger numbers of species of wild vegetables and culinary herbs? The adjective 'culinary' is needed as the green parts of plants have been used in Poland widely, mainly as infusions, in a medicinal context [6]. It must be stressed that the occurrence of culinary aherbia/herbophilia and mycophobia/mycophilia is not necessarily correlated. Poles are strongly mycophilous [33] and 'fructophilous' but have historically largely neglected the use of green vegetables and culinary herbs, whereas other national cultures exert different patterns, an issue which needs further study. Utilisation of a large number of species of green vegetables is a characteristic feature of agricultural communities, particularly those in which food shortages are frequent. In such societies the utilization of weeds as food provided extra calories and made space for the growth of main crops. Once the danger of famine is removed, some societies reject green food as a symbol of famine, others preserve at least some of the "famine" vegetables as traditional foods or food additives.

The possible reasons why Poles have used few green parts of wild species have already been pointed out by Łuczaj & Szymański [1]. Most edible plant species in the Mediterranean are used as spices, salads or appetisers, not as staple foods [27,30]. Many of those which are found as common plants in Poland (e.g. *Thymus *spp.), have hardly ever been used in Polish cooking as spices, although they are often listed by ethnographic sources as medicinal plants throughout Poland [6]. The primary reason for the difference in attitudes towards herbs between Poland and the Mediterranean may be climate. In warmer climates the addition of herbs to meats, dairy and sauces kept them from going off, whereas in the Polish temperate climate there is less need for this. Hence "pure", refined foods like white sugar, white bread and pure good quality meat were most highly prized, and wild plants, apart from fruits and mushrooms, were associated only with times of famine and seasonal spring food shortages. Another reason is that Poland, a mostly flat country with reliable rainfall, has been a thoroughly agricultural country with a large proportion of arable land, where vegetables could be easily cultivated, whereas the countries of the Mediterranean Basin are very mountainous, with a large proportion of land covered by stony semi-arid pastures where cultivation of vegetables is difficult and wild plants could have been a valuable addition to the pastoral economy.

### Not only Carpathians

The results of this study undermine the conclusions of a recent review of edible plants of Poland [1], which stated that many more wild edible plants have been collected in the Carpathians, particularly in their western part, than in lowland Poland. Now it can be clearly seen that such results were caused by the considerably larger number of ethnographic studies undertaken in the Carpathians, since this study proves that places where large numbers of edible plants were collected existed in a few parts of Poland in the mid-20^th ^century (Fig. 2), not only in the Carpathians, but along the eastern border as well as in Polish communities originating from the expanded Soviet Union (Lithuania, Ukraine and particularly Belarus), which had been resettled to the west of Poland. We can imagine the enclaves in which traditional knowledge about edible plants persisted as less-developed rural areas within biodiverse regions, or as families particularly oriented towards gathering. The high heterogeneity of species richness of the plant inventories obtained may have been caused by individual differences among informers, differences between locations and differences in the effort put in by the researchers to obtain information.

The longitudinal pattern observed in species richness of the studied questionnaires is typical of many ethnographic phenomena in Poland [2,3]. The west of Poland was strongly influenced by Germany, its industry and modernisation of farming practices, remaining under Prussian occupation from the end of the 18^th ^century until 1918. The east and the south constituted the outskirts of the Russian and the Austro-Hungarian Empires and were less affected by modernisation. Hence more traditional folk culture was preserved in the eastern part of Poland.

### Present state of gathering wild food plants

Since 1948, a gradual decrease in the use of wild food plants has occurred in Poland and the data presented here are of historical character. At the time of data collection (1948) people still remembered the use of some famine plants, the soup of *Rumex acetosa *leaves was made in nearly every village and wild fruit preserves for winter were made in a large proportion of households in the countryside. As recent studies by the team of Polish Ethnographic Atlas showed [31], at the beginning of the 21st century the gathering of wild food plants has become restricted to a few individuals particularly interested in this kind of activity. In Jędrusik's thesis [31], data on the gathering of wild plants and mushrooms from 82 villages, in 1964–68 and in 2000–2003, obtained using similar questionnaires, were compared. The number of villages where they were collected in 2000–2003 had decreased to a fraction of the number in the 1960s (e.g. for *Vaccinium myrtillus *from 77 to 21, *Armoracia rusticana *from 67 to 17, *Rumex acetosa *from 59 to 19). The gathering of some species had stopped entirely (e.g. *Prunus spinosa *or *Vaccinium uliginosum*). In contrast to these data the frequency of mushroom collection has not changed much in the last few decades, apart from a change in the way they are stored for winter (a shift from drying towards pickling and freezing).

## Conclusion

1. Fruits were the most frequently utilised group of wild plants. Green parts of plants, although also frequently recorded were, apart from *Rumex *spp. treated mainly as famine food or children's snacks.

2. Children were the most important collectors and users of wild plants.

3. The number of edible plants used was similar, and relatively low, in all regions of Poland. It was on average slightly lower in the area where strong migrations from the expanded Soviet Union occurred after World War II than in the part of Poland where few migrations occurred after World War II, however the difference was not significant.

4. The identification of herbarium specimens clarified many uncertainties concerning folk taxa reported in earlier literature and confirmed the use of species previously not reported from Poland.

## Competing interests

The author(s) declare that they have no competing interests.

## Appendix

### Identified taxa recorded in Questionnaires 1 and 2

Each entry has the following format:

Latin name 'H' + number of identified herbarium specimens (number of recorded localities + 'loc.') – parts used, preparation methods; list of localities for some interesting species; LN: all recorded local names (starting from the commonest).

Each locality is expressed as: "village name (nearest town name, region code)". Regions (*województwo*) were coded as follows:

Sl – śląskie, Mp – małopolskie, Pk – podkarpackie, Lu – lubelskie, Sw – świętokrzyskie, Łd – łódzkie, Mz – mazowieckie, Ps – podlaskie, Wm – warmińsko-mazurskie, Pm – pomorskie, Kp – kujawsko-pomorskie, Wp – wielkopolskie, Zp – zachodniopomorskie, Ls – lubuskie, Ds – dolnośląskie, Op – opolskie.

#### Aceraceae

***Acer platanoides *L. **H1 (3 loc.) – sap, raw; Oleksin (Bielsk Podlaski, Ps); Biesal (Olsztyn, Wm); Radowo Małe (Łobez, Zp); LN: klon.

***Acer pseudoplatanus *L. **H0 (1 loc.) – sap, raw; Szaflary (Nowy Targ, Mp); LN: jawor.

#### Apiaceae

***Carum carvi *L. **H3 (19 loc.) – seeds, as spice in bread and sauerkraut; LN: kminek, kmin, chminek, karolek, karólek, karulik.

***Heracleum sphondylium *L. **H1 (1 loc.) – green parts, as famine vegetable and soup before World War I; Rabka Zdrój (Nowy Targ, Mp); LN: burak dziki, borszczek.

***Pastinaca sativa *L. **H0 (4 loc.) – roots; Nasutów (Lublin, Lu); Wola Osowińska (Radzyń Podl., Lu); Glinnik (Tomaszów Maz., Łd); Zamość (Piotrków Tryb., Łd); LN: pasternak.

#### Araceae

***Acorus calamus *L. **H1 (13 loc.) – raw young shoots as children's snack; LN: tatarak, kalmus, tatarczok.

#### Asteraceae

***Bellis perennis *L. **H0 (1 loc.) – as salad, still occasionally used in 1948, but more common in times of famine; Pruchna (Cieszyn, Sl); LN: stokrótka, gęsie pómpki.

***Carlina acaulis *L. **H1 (1 loc.) – "flowers and fruits"; Rabka Zdrój (Nowy Targ, Mp); LN: oset, dziewięćsił, dziewięciornik.

***Centaurea cyanus *L. **H3 (12 loc.) – petals used by children to make a fermented drink with sugar; LN: chaber, bławat, bławatek, modrak, modrok, głowoc.

***Chamomilla recutita *(L.) Rauschert **(syn.* Matricaria chamomilla *L.) H0 (5 loc.) – shoots, for infusion; LN: rumianek, kamelek.

***Cirsium *spp. **H2: ***C. arvense *(L.) Scop. **H1, ***C. rivulare *All. **H1 (5 loc.) – leaves used in boiled and fried dishes, only in times of famine; *C. arvense*: Płaskocin (Łowicz, Łd); *C. rivulare*: Biały Dunajec – Stołowe (Zakopane, Mp); *Cirsium *sp.: Brzeźnica Bychawska (Lubartów, Lu), Przerośl (Suwałki, Lu), Radowo Małe (Łobez, Zp); LN: oset.

***Taraxacum *spp. **H2 (10 loc.) – leaves, raw, "served like lettuce"; LN: mlecz, moik, mlecznik, mniszek lekarski, moiczek, dmuchawiec.

***Tragopogon pratensis *L. s.l. **H1 (1 loc.) – stalks, chewed by children and adults and then spat out; Frydman (Nowy Targ, Mp); LN: kozibroda.

#### Berberidaceae

***Berberis vulgaris *L. **H1 (4 loc.) – fruits, raw and preserved, used to make vinegar; LN: berberys.

#### Betulaceae

***Alnus *sp. **H0 (1 loc.) – "alder bark", ground, added to famine bread in earlier times; Sonina (Łańcut, Pk); LN: olcha.

***Betula *spp. **H5: ***Betula pendula *Roth **(syn. *B. verrucosa *Ehrh) H4 &***B. pubescens *Ehrh. **H1 (26 loc.) – sap, mainly as children's beverage, raw; in Rabka Zdrój (Nowy Targ, Mp) also bark of young trees; LN: brzoza.

#### Boraginaceae

***Symphytum officinale *L. **H2 (3 loc.) – nectar from flowers as children's snack, leaves for famine soups; soup called *szabaga*: Pantalowice (Przeworsk, Pk); flowers: Kalnica (Bielsk Podl., Ps), Przerośl (Suwałki, Ps); LN: żywokost, miodownik.

#### Brassicaceae

***Armoracia rusticana *P. Gaertn., B. Mey. & Scherb. **(syn.* Armoracia lapathifolia *Gilib., *Cochlearia armoracia *L.) H3 (26 loc.) – grated roots used as meat condiment or side-dish; LN: chrzan, krzan, krzun, krzon.

***Capsella bursa-pastoris *(L.) Medik. **H0 (1 loc.) – fruits, as children's snack; Kórnik (Poznań, Wp); LN: tasznik, świętojański chleb.

***Sinapis *sp. **or ***Raphanus *sp. **H0 (4 loc.) – seeds, leaves, fried; LN: psconak, gorczyca, ognicha.

#### Cannabaceae

***Humulus lupulus *L. **H0 (4 loc.) – flowers, dried, added to beer; LN: chmiel.

#### Caprifoliaceae

***Sambucus nigra *L. **H5 (24 loc.) – fruits, preserved as juice, jam or wine; in E Poland fruits also cooked in soups; flowers, dried for infusions; names of soups – *bzianka*: Suleje (Łuków, Lu), Radomyśl (Siedlce, Mz), *czernina*: Wola Osowińska (Radzyń Podl., Lu), *barszcz*: Brzeźnica Bychawska (Lubartów, Lu); LN: bez dziki, bez czarny, holunder, besk.

***Viburnum opulus *L. **H0 (8 loc.) – fruits, in Szaflary (Nowy Targ, Mp) used for juice, no details of use in other localities; LN: kalina.

#### Chenopodiaceae

***Chenopodium album *L. **H16 (50 loc.) – leaves, boiled and fried (potherbs, soups); LN: lebioda, łoboda, komosa, lebida, łapucha, łopucha, kumosa, warmuz, jarmucha, jarmuż, jarmużka, faćka, bańdocha, zielenina.

#### Convolvulaceae

***Convolvulus arvensis *L. **H1 (1 loc.) – shoots, boiled, past use; Rabka Zdrój (Nowy Targ, Mp); LN: powójka.

#### Corylaceae

***Corylus avellana *L. **H9 (29 loc.) – raw nuts, sometimes dried for use at Christmas; LN: leszczyna, orzech laskowy, orzech leskowy, lescyna, lyska, oreche mały, orzech leśny.

#### Cupressaceae

***Juniperus communis *L. **H0 (7 loc.) – fruits, no more data; LN: jałowiec.

#### Equisetaceae

***Equisetum arvense *L. **H2 (7 loc.) – young shoots called "szypułki" and underground bulbils eaten raw by children; shoots: Szaflary (Nowy Targ, Mp), Biały Dunajec – Stołowe and Poronin (Zakopane, Mp); bulbils: Radowo Małe (Łobez, Zp); Oleksin (Bielsk Podl. Ps), Boża Wola (unidentified location mentioned in the list for Pepłowo, Mz); Unknown Part: Pantalowice (Przeworsk, Pk); LN: skrzyp, szypułka, chrząstka, chwoszczka.

#### Ericaceae

***Vaccinium myrtillus *L. **H9 (47 loc.) – fruits, raw and preserved, as juice or jam, used for soups, also dried; LN: (czarna) jagoda, borówka czarna, czernica, sinicy.

***Vaccinium oxycoccos *L. **(syn. *Oxycoccus palustris *Pers.) H1 (9 loc.) – fruits, usually in jams and sauces; in Przerośl (Suwałki, Ps) large quantities added to sauerkraut or, more rarely, cooked and thickened with flour (so called *kisiel*); LN: żurawina, żurawie, żurosie, zórowie.

***Vaccinium uliginosum *L. **H2 (10 loc.) – fruits, raw or as juice, jam, soup or compote; LN: włochynia, łochynia, łochina, łochinia, mochynia, ochynia, wołochy, pijanica, zajęcza jagoda.

***Vaccinium vitis-idaea *L. **H7 (34 loc.) – fruits, raw or as juice, jam, soup or compote; a few informers complained that use was decreasing due to the high price of the sugar needed for preserving it; LN: borówka (czerwona), borówka, brusznica, bruśnica, borówka jesienna, barszczownik.

#### Fabaceae

***Robinia pseudoacacia *L. **H2 (7 loc.) – flowers, raw, also in jams (Oborniki, Wp) and cakes in Nowe Kramsko (Zielona Góra, Lb); LN: akacja, akacki, myszki.

***Trifolium *spp. **5: ***T. pratense *L. **H2, ***T. repens *L. **H2, ***T. montanum *L. **H1 (13 loc.) – nectar from flowers sucked by children; LN: koniczyna, konicz, krasikoń, rosikoń.

***Vicia *spp. **sp H0 (1 loc.) – whole fruits, probably immature; Frydman (Nowy Targ, Mp); LN: wyka.

#### Fagaceae

***Fagus sylvatica *L. **H1 (9 loc.) – raw nuts eaten by children; LN: buk, buczyna, bukwia.

***Quercus robur *L. **H1 (12 loc.) – acorns, roasted to make coffee; galls (called *jabłka z dębu*) were used to make a beverage in Barwino (Słupsk, Pm); LN: dąb, żołędź, żołądź.

#### Grossulariaceae

***Ribes spp.: Ribes nigrum *L. **H1 &*** R. spicatum *Robson **(syn. *R. schlechtendalii *Lange) H3 (11 loc.) – fruits, raw or preserved covered in sugar or as juice and jam; most reports come from E Poland (Podlasie and Lublin area); LN: porzeczka, świętojanki, for *R. nigrum *also: smurodina, smrodina, jagody samorodyn, bździuchi.

***Ribes uva-crispa *L. **(syn. *R. grossularia *L.)H0 (1 loc.) – no more data; Słosinko and Falkowice (Bytów, Pm); LN: angryst, agrest.

#### Hydrocharitaceae

***Stratiotes aloides *L. **H2 (3 loc.) – boiled leaves and roots, important famine food in the 19th century; Glinnik (Tomaszów Maz., Łd), Zamość (Piotrków Tryb., Łd), Zarzęcin (Opoczno, Łd); LN: kozieł, koziełek, koziołek.

#### Lamiaceae

***Glechoma hederacea *L. **H1 (1 loc.) – leaves and shoots, fried or added to soups, also dried for future use; Rabka Zdrój (Nowy Targ, Mp); LN: kurdybanek, urbanek, bluszczyk.

***Lamium album *L. **H1 (3 loc.) – nectar from flowers sucked by children in (Gruszów (Proszowice, Mp), Trześniów (Brzozów, Pk), Pantalowice (Przeworsk, Pk); in Pantalowice flowers also dried for tea and whole shoots formerly used for famine soup; LN: martwa pokrzywa, głucha pokrzywa, dzika pokrzywa.

***Mentha *spp. **H4, including ***M. arvensis *L. **H1 and ***M. longifolia *(L.) Hudson. **(syn. *M. longifolia *(L.) L. H2 (11 loc.) – shoots, in tea, soups and dumplings; *M. arvensis*: in Przerośl (Suwałki, Ps), infusion; *M. longifolia*: Rabka Zdrój (Nowy Targ, Mp), in soups, Berejów (Lubartów, Lu), in soups and dumplings; LN: mięta, miętka.

***Stachys palustris *L. **H1 (1 loc.) – rhizomes, eaten raw, dried or powdered into flour added to soups, formerly famine food, then as children's snack; Biały Dunajec – Stołowe (Nowy Targ, Mp); LN: dzika marchew.

***Thymus *spp.: *T. pulegioides *L**. H2, ***T. serpyllum *L. **H1 (5 loc.) – whole flowering plants, infusion or as a spice; *T. pulegioides *as a spice or infusion used by indigenous inhabitants of Wałcz (Zp), only as infusion in Przerośl (Suwałki, Ps); *T. serpyllum *as infusion, in Barwino (Miastko, Pm); LN: macierzanka, cząberek.

#### Liliaceae

***Allium *sp. **H2 (2 loc.) – aerial bulbils, as spice; Oleksin (Bielsk Podlaski, Ps); Oborniki (Wp); LN: dziki czosnek, czosnyk polny.

***Maianthemum bifolium *(L.) F.W. Schmidt **(syn. *Majanthemum bifolium *(L.) DC.) H1 (4 loc.) – raw, as children's snack; Drelów (Biała Podl., Lu); Nasutów (Lublin, Lu); Barwino (Słupsk, Pm); Dalechowice (Kazimierza Wlk., Sw); LN: konwalijka, ptasie wino, ptasie winko, winogron dziki, winogron lasowy.

#### Loranthaceae

***Viscum album *L. **H1 (1 loc.) – fruits, commonly eaten raw by children and adults, sought after during tree felling; Zarzęcin (Opoczno, Op); LN: jemioła, imioła.

#### Malvaceae

***Malva *spp. **H5: ***M. sylvestris *L. **H3 &***M. neglecta *Wallr. **(syn. *M. rotundifolia *L.) H1 (9 loc.) – immature fruits, eaten raw, mainly in western Poland; *M. neglecta: *Barwino (Słupsk, Pm), *M. sylvestris: *Oborniki (Wp), Maciowakrze (Kędzierzyn-Koźle, Op), Nowe Kramsko (Zielona Góra, Lb); LN: ślaz, świętojański chleb, Boży chleb, babi chleb, bobki, rajski chleb, gomółki, kopytnik, babka, chleb.

#### Oxalidaceae

***Oxalis *spp**.: H11, ***O. acetosella *L. **H9, ***O. stricta *L. **s.l. H2 (31 loc.) – as children's snack, some memory left of its former use in soups; LN: zajęcza kapusta, zajęczy szczaw, szczaw zazuli, zajęcy scow, zajęcy scaw, zajęcza koniczynka, koniczyna zającowa, rosikon, kukułczy szczaw, kapusta dzika, kapusta zająca, kapusta zajęca, szczaw (zajęczy), szczawik zajęczy.

#### Papaveraceae

***Papaver rhoeas *L. **H0 (1 loc.) – seeds, no more details; Oleksin (Bielsk Podl., Ps); LN: dziki mak, patruch.

#### Pinaceae

***Picea abies *(L.) Karsten **(syn. *P. excelsa *(Lam.) Link.) H0 (1 loc.) – cambium; Frydman (Nowy Targ, Mp); LN: świerk.

***Pinus sylvestris *L. **H0 (1 loc.) – young shoots; Oleksin (Bielsk Podl., Ps); LN: sosna.

#### Plantaginaceae

***Plantago lanceolata *L. **H0 (1 loc.) – leaves; Skoczów (Cieszyn, Sl); LN: babka lancetowata.

#### Poaceae

***Bromus secalinus *L. **H0 (1 loc.) – probably fruits, formerly used as famine food; Włosań (Kraków, Mp); LN: stokłosa.

***Elymus repens *(L.) Gould **(syn. *Agropyron repens *(L.) P. Beauv., *Triticum repens *L.) H4 (19 loc.) – only as famine food until the beginning of the 20^th ^century, rhizomes, dried, ground and made into flour used to make bread or thick soup; LN: perz, pyżowica.

***Glyceria *sp. **H0 (3 loc.) – grains; Grochówka (Siedlce, Mz), Barwino (Słupsk, Pm), Kórnik (Poznań, Wp); LN: manna, pływka.

***Phleum pratense *L. **H1 (1 loc.) – seeds, ground into flour to make bread, probably in the past as famine food; Włosań (Kraków, Mp); LN: regras, tymotka.

#### Polygonaceae

***Polygonum lapathifolium *L. ssp. *pallidum ***(syn. *P. tomentosum *Schrank) H2 and related taxa (3 loc.) – leaves, as famine food (potherb); P. lapathifolium: Barwino (Słupsk, Pm), Zarzęcin (Opoczno, Łd); Polygonum sp.: Zamość (Piotrków Tryb., Łd); LN: rdest, grdes, rdes, gdest, pliskucha.

***Rumex obtusifolius *L. **H1 (1 loc.) – leaves for compotes; Maciowakrze (Kędzierzyn-Koźle, Op); LN: szczaw koński.

***Rumex *spp. **(arrow-leaved species only) H25: ***R. acetosa *L. **H16, ***R. acetosella *L. **H4, ***R. thyrsiflorus *Fing. **H1 (75 loc.) – eaten raw by children or cooked in soup and compotes; LN: szczaw, kwasielec, kwasek, baśka, kwaśne listka, tczaw, kwaśna kapusta, zajęcza kapusta, scyk, ścow, śczoch, ściow, szczołw, szczowik, kwaśniarka, kwasyk, kwaśne listka, kwaśna kapusta, polna kapusta, szczaw wróblęczy.

#### Polypodiaceae

***Polypodium vulgare *L. **H1 (3 loc.) – rhizomes, as children's snack; Frydman and Szaflary (Nowy Targ, Mp), Mszana Dolna (Limanowa, Mp); LN: paproć, paprótka.

#### Primulaceae

***Primula elatior *(L.) **Hill H0 (1 loc.) – flowers; Skoczów (Cieszyn, Sl); LN: pierwiosnki, kluczyki.

#### Ranunculaceae

***Ranunculus *sp. **(probably ***R. repens *L. **or ***R. ficaria *L.**) H0 (1 loc.) – leaves; Skoczów (Cieszyn, Sl); LN: jaskier niski.

#### Rhamnaceae

***Frangula alnus *Miller **H0 (3 loc.) – fruits, in Brzeźnica Bychawska made into jams, no data on preparation in other locations; Brzeźnica Bychawska (Lubartów, Lu); Barwino (Słupsk, Pm); Radowo Małe (Łobez, Zp); LN: kruszyna.

#### Rosaceae

***Crataegus *spp. **H7, including ***C. monogyna ***Jacq. H6 (14 loc.) – eaten raw by children or more rarely made into wine and marmalade; LN: głóg, babiorka, barania gruszka, rajskie jabłka, mączki, kowalowe kluski, srót.

***Crataegus *spp. **or ***Rosa *spp. **– imprecisely identified plants mentioned as *głóg *or *gug *(16 loc.) – fruits.

***Fragaria vesca *L. **H11 (47 loc.) – fruits, raw and preserved; leaves, dried to make tea; LN: poziomka, czerwona jagoda, podzimka, jagoda cerwuno, pozymka, pożymki, poziemka.

***Malus *spp. **H0 (4 loc.) – fruits; LN: dzikie jabłka, lesionki, płonka.

***Prunus padus *L. **(syn. *Padus avium *Mill.) H2 (11 loc.) – fruits, raw as children's snack; LN: czeremcha, czeremucha, huciapa, kocierpka, korcipka, korciepka, korczyna.

***Prunus spinosa *L. **H5 (27 loc.) – fruits, raw as children's snack and preserved as juice, jam or wine; LN: tarnina, tarki, torki, ciarki, ciorki, kocierypka, tarcyna, ciernie, dzika śliwka.

***Pyrus *spp. **H0 (5 loc.) – fruits, raw; LN: dzikie gruszki, gruski, gniłki.

***Rosa *spp. **mainly ***R. canina *L. **H5 (21 loc.) – fruits, raw, also preserved as jam and wine; LN: dzika róża, głóg, polna róża, szyp, koralina, supsyna.

***Rubus idaeus *L. **H7 (32 loc.) – fruits, raw or made into juice and preserves; leaves, dried to make tea; LN: malina.

***Rubus saxatilis *L. **H0 (5 loc.) – fruits, no more data; LN: kamionka, kęmiunka, kamiuszczka, probably also: podmalina.

***Rubus *subgenus *Rubus *spp. **H13: ***R. caesius ***H6, ***Rubus *section *Rubus ***H7 (48 loc.) – fruits, raw, also preserved as jam, juice and wine; LN: jeżyna, jażyna, izyna, oryna, dziady, czernice, ostrężnice, ostrężyna, jagody kolkowe, cierniówki, czarnicy, malina czarna, bostrążyny, popieliny.

***Sedum maximum *(L.) Hoffm. **(syn. *S. telephium *L.) H1 (1 loc.) – thick roots; Nowe Kramsko (Zielona Góra, Lb); LN: zajace pyry.

***Sorbus aucuparia *L. emend. Hedl. **H1 (9 loc.) – fruits, juice, jam, soup, liqueurs; LN: jarzębina, jarząb.

#### Rubiaceae

***Galium odoratum *(L.) Scop. **(syn. *Asperula odorata *L.) H1 (1 loc.) – dried flowering plants used by autochtonous Mazurians as spice in desserts, beverages and liqueurs, Jagodzin (Pisz, Wm); LN: mistrz leśny, waldmeister.

#### Salicaceae

***Salix *sp. **H0 (1 loc.) – probably catkins; Trześniów (Brzozów, Pk); LN: wierzbina.

#### Tiliaceae

***Tilia *spp. **(all herbarium specimens belong to *Tilia cordata *Miller, although *T. platyphyllos *Scop. may be used as well) H2 (17 loc.) – infusion of flowers; in Przerośl (Suwałki, Ps) young bark was eaten by children; LN: lipa.

#### Ulmaceae

***Ulmus *spp. **H0 (1 loc.) – fruits and leaves; Drelów (Biała Podl., Lu); LN: wiąz.

#### Urticaceae

***Urtica ***H13: ***Urtica dioica *L. **H9 &*** U. urens *L. **H4 (38 loc.) – young leaves, cooked or fried, as potherb, soup, or as an addition to scrambled eggs, still used but strongly decreasing, perceived as poverty food; LN: pokrzywa, żegawka, zykawka, żgawka, żagawka, żgawka, żagiewka, zygawka.

#### Violaceae

***Viola hirta *L. **H1 (1 loc.) – sweet roots eaten by children; Mszana Dolna (Limanowa, Mp); LN: słodki korzeń.

***Viola tricolor *L. **H1 (1 loc.) – flowers for infusion, dried for winter; Bielice (Nysa, Op); LN: fiołek polny, fiołek dziki.

#### Vitaceae

***Parthenocissus *sp. **H1 (*P. inserta *(A. Kerner)Fritsch or *P. quinquefolia *(L.)Planchon) (1 loc.) – fruits, collected by children to make wine; Wólka Rudnicka (Kraśnik, Lu); LN: dzikie wino.
